# Structural changes, thermodynamic properties, ^1^H magic angle spinning NMR, and ^14^N NMR of (NH_4_)_2_CuCl_4_·2H_2_O

**DOI:** 10.1039/c8ra00170g

**Published:** 2018-02-09

**Authors:** Ae Ran Lim, Sun Ha Kim

**Affiliations:** Analytical Laboratory of Advanced Ferroelectric Crystals, Jeonju University Jeonju 55069 South Korea aeranlim@hanmail.net arlim@jj.ac.kr +82-63-220-2053 +82-63-220-2514; Department of Science Education, Jeonju University Jeonju 55069 South Korea; Seoul Western Center, Korea Basic Science Institute Seoul 03759 South Korea; Department of Chemistry, Kyungpook National University Daegu 41566 South Korea

## Abstract

The structural changes and thermodynamic properties of (NH_4_)_2_CuCl_4_·2H_2_O were studied by differential scanning calorimetry (DSC) and thermogravimetric (TG) analysis. In addition, the chemical shift, line width, and spin-lattice relaxation time of the crystals were also investigated by ^1^H magic angle spinning nuclear magnetic resonance (MAS NMR), focusing on the role of NH_4_ and H_2_O near the phase transition temperature. The change at *T*_C2_ (=406 K) and *T*_C3_ (=437 K) seems to be a chemical change caused by thermal decomposition rather than a physical change such as a structural phase transition. The changes in the temperature dependence of these data near *T*_C2_ are related to variations in the environments surrounding NH_4_ and H_2_O. The ^14^N NMR spectrum is also measured in order to investigate local phenomena related to the phase transition.

## Abstract 2

The structural changes and thermodynamic properties of (NH_4_)_2_CuCl_4_·2H_2_O were studied by differential scanning calorimetry (DSC), thermogravimetric (TG) analysis, and nuclear magnetic resonance (NMR).

## Graphical abstract

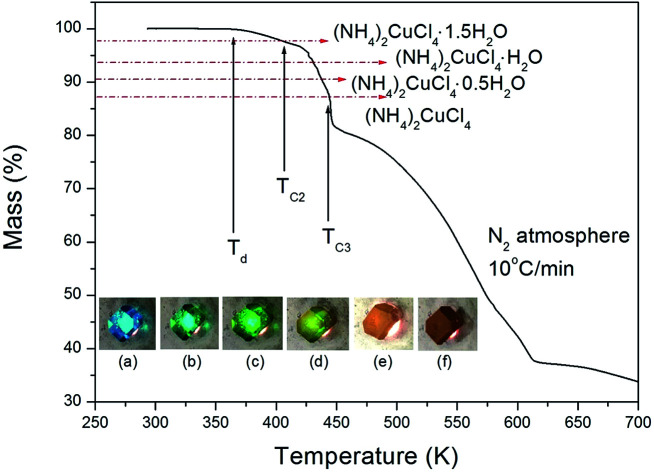

## I. Introduction

**Fig. 1:**
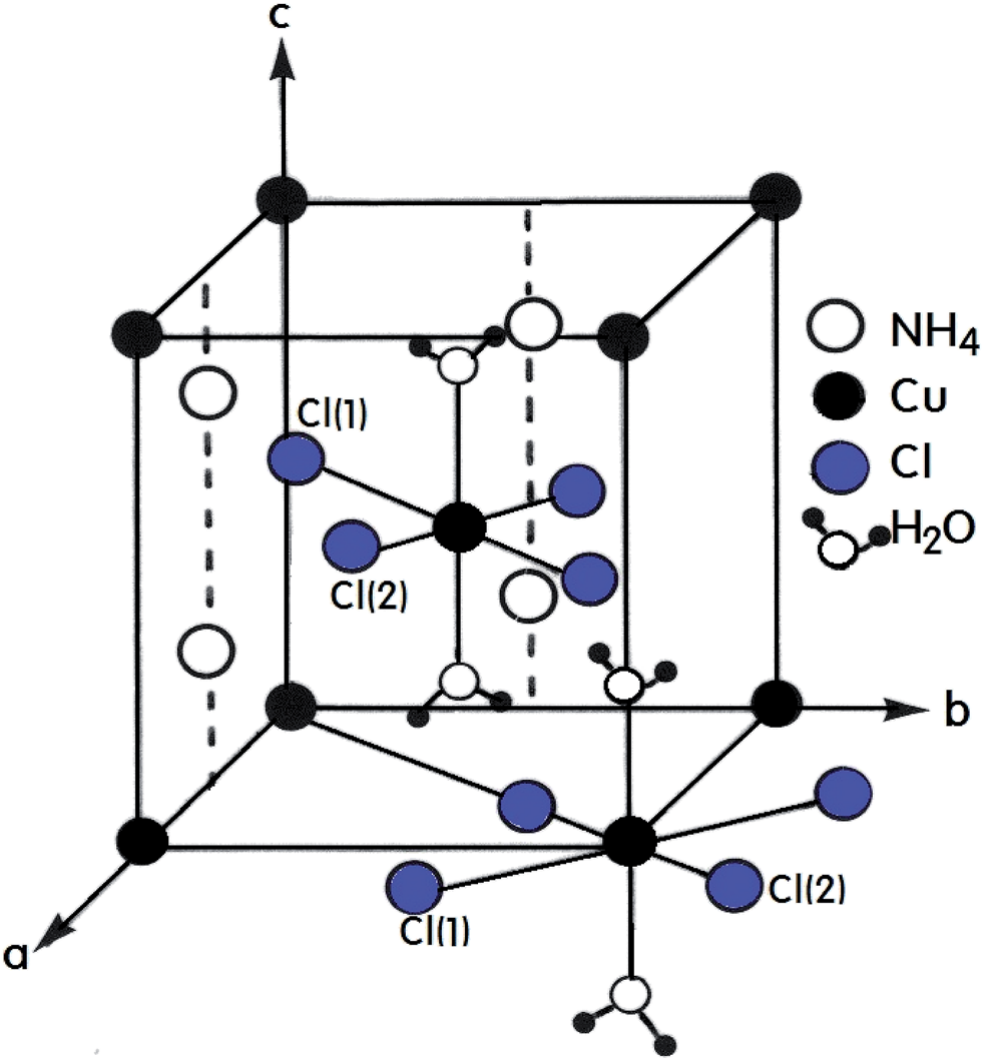
Tetragonal structure of the (NH_4_)_2_CuCl_4_·2H_2_O crystal.

A number of compounds with the chemical formula A_2_BX_4_·2H_2_O, where A = NH_4_, K, Rb, Cs and B = Cu, Mn, Ca, Ni are monovalent and divalent metal ions, respectively, and X = Cl, Br is a halide ion, crystallize as perovskite-type two-dimensional layered structures.^1–7^ Crystals with this arrangement can be divided into two classes according to their symmetry and structure.^8–10^ The first class includes compounds containing Cu^2+^ ions that crystallize with tetragonal symmetry with the space group *P*4_2_/*mnm* at room temperature. The tetrahedrons surrounding the divalent metal ions placed at the corners of the unit cell are rotated by exactly 90° with respect to the tetrahedron surrounding the ion at the center of the cell. The A^+^ ions are placed in the almost cubic cavities formed by the tetrahedrons.^11,12^ Crystals containing Mn^2+^, Ca^2+^, and Ni^2+^ ions form the second of these two classes with triclinic symmetry and the space group *P*_1_. The (NH_4_)_2_CuCl_4_·2H_2_O crystal, which is an example of the former, exhibits a structural phase transition from the point group 4/*mmm* to the point group 4(bar)2*m* at 200 K.^13–15^ In addition, two different phase transitions at approximately 383 K and 413 K were observed in the DC electric conductivity measurements reported by Narsimlu *et al.*^16^ At room temperature, (NH_4_)_2_CuCl_4_·2H_2_O forms a tetragonal structure with space group *P*4_2_/*mnm*, as shown in Fig. 1.^14^ The unit cell contains two formula units and has the lattice constants *a* = *b* = 7.596 Å, *c* = 7.976 Å.^17^ The unit cell contains two Cu^2+^ ions at equivalent positions (0, 0, 0) and (1/2, 1/2, 1/2) each surrounded by an approximate octahedron of four Cl^−^ ions and two water molecules. The line connecting the water molecules is parallel to the crystallographic *c*-axis.^1^ The water molecule is trigonal coordinated and forms two equivalent O–H⋯Cl(1) hydrogen bonds with an H⋯Cl(1) length of 2.186 Å. The O–H distance in the water molecule is 0.965 Å. The NH_4_^+^ is hydrogen bonded to the Cl(1) atoms at 3.357 Å, while in the other orientation, it is bonded to the Cl(2) atoms at 3.370 Å. The respective N–H distances are 1.011 Å and 1.021 Å.^17^

The purpose of this study is to investigate the structural changes and thermodynamic properties of (NH_4_)_2_CuCl_4_·2H_2_O single crystals using differential scanning calorimetry (DSC) and thermogravimetric (TG) analysis. Further, the chemical shift, line width, and spin-lattice relaxation time *T*_1ρ_ in the rotating frame of (NH_4_)_2_CuCl_4_·2H_2_O are measured by ^1^H magic angle spinning/nuclear magnetic resonance (MAS/NMR) near the phase transition temperature, focusing on the role of NH_4_ and H_2_O. In addition, the ^14^N NMR spectrum in the laboratory frame is measured as a function of the temperature, in an attempt to understand the structural geometry near the phase transition temperature. Our findings represent the first report on the thermodynamic properties and NMR characteristics of (NH_4_)_2_CuCl_4_·2H_2_O, and are useful for understanding the phase transitions.

## II. Experimental method

Single crystals of (NH_4_)_2_CuCl_4_·2H_2_O were grown by slowly evaporating aqueous solutions containing a stoichiometric mixture of NH_4_Cl and CuCl_2_·2H_2_O in the molar ratio of 2 : 1 at room temperature. The obtained crystals were light blue. The phase transition temperature was determined using a Dupont 2010 DSC instrument. The rate of temperature change during heating was 10 °C min^−1^.

The ^1^H MAS NMR spectra of (NH_4_)_2_CuCl_4_·2H_2_O in a rotating frame were obtained using the Bruker DSX 400 FT NMR spectrometer at the Korea Basic Science Institute, Western Seoul Center. The static magnetic field used was 9.4 T, and the central radio frequency was set at *ω*_0_/2π = 400.13 MHz for the ^1^H nucleus. The powder sample was placed in a 4 mm MAS probe, and the MAS rate was set to 5 kHz to minimize spinning sideband overlap. The spin-lattice relaxation times in the rotating frame were measured using a saturation recovery pulse sequence called sat–*t*–π/2; the nuclear magnetizations of the ^1^H nuclei at time *t* after the sat pulse, a combination of one hundred π/2 pulses applied at regular intervals, were determined following the π/2 excitation pulse. The width of the π/2 pulse was 3.45 μs below 410 K and 6.7 μs above 420 K for ^1^H.

In addition, the ^14^N NMR spectra of the (NH_4_)_2_CuCl_4_·2H_2_O single crystals in the laboratory frame were measured using a Unity INOVA 600 NMR spectrometer at the Korea Basic Science Institute, Western Seoul Center. The static magnetic field was 14.1 T, and the Larmor frequency was set to *ω*_0_/2π = 43.342 MHz. The ^14^N NMR experiments were performed using the solid-state echo sequence: 3.7 μs–*t*–3.7 μs–*t*. The NMR measurements were obtained in the temperature range of 180–430 K. Unfortunately, the chemical shift and resonance frequency could not be measured above 430 K because the NMR spectrometer did not have adequate temperature control at high temperature. The temperatures of all the samples were maintained at constant values by controlling the helium gas flow and heater current, which yielded an accuracy of ±0.5 K.

## III. Results and discussion

**Fig. 2:**
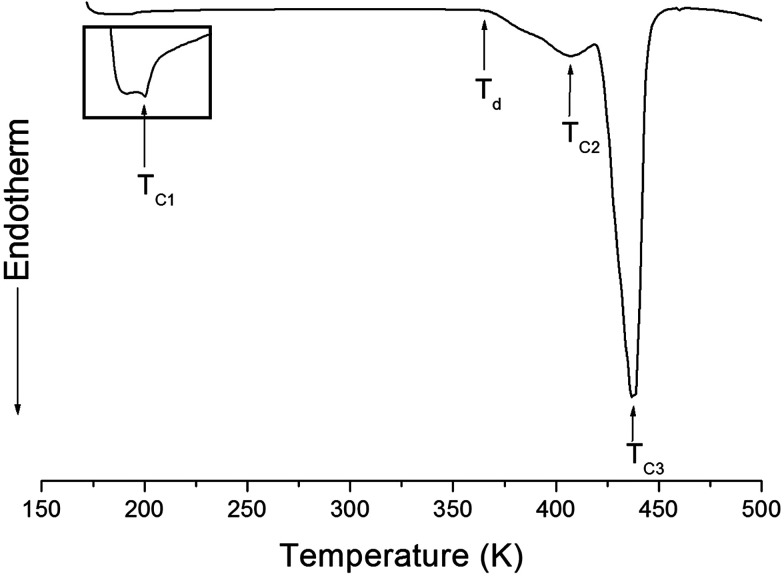
DSC thermogram of (NH_4_)_2_CuCl_4_·2H_2_O upon heating.

**Fig. 3:**
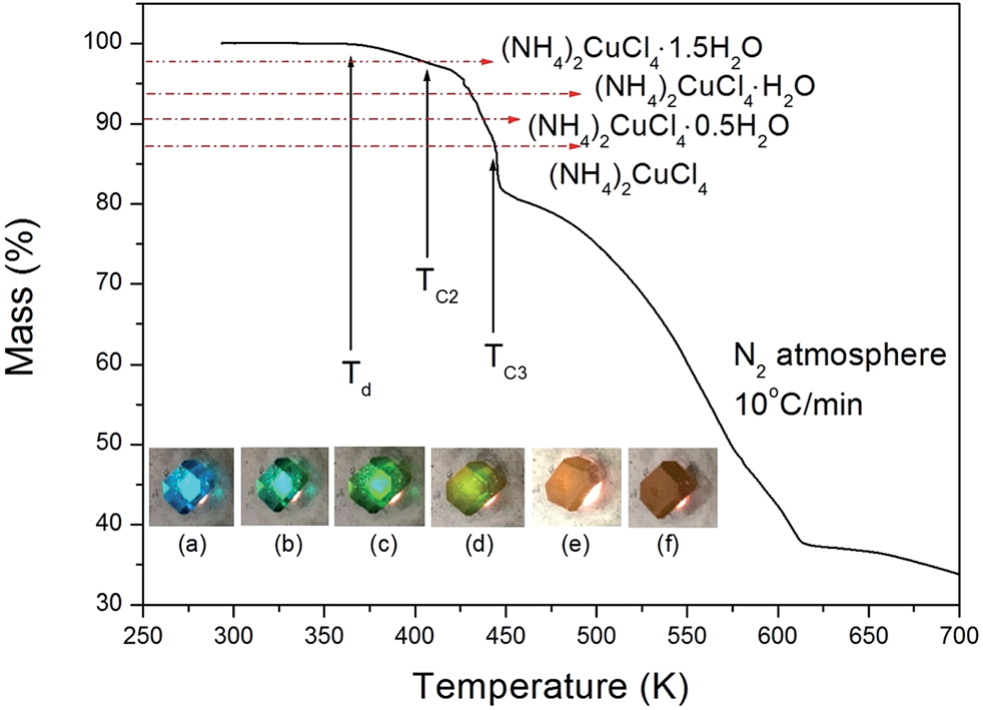
Thermogravimetric analysis (TGA) of (NH_4_)_2_CuCl_4_·2H_2_O (inset: color changes according to the temperature: (a) 295 K, (b) 373 K, (c) 386 K, (d) 393 K, (e) 433 K, and (f) 448 K).

The structure of the (NH_4_)_2_CuCl_4_·2H_2_O crystals at room temperature was determined by X-ray diffraction (XRD) (PANalyical, X'pert Pro MPD) with a Cu-Kα (*λ* = 1.5418 Å) radiation source at the Korea Basic Science Institute, Western Seoul Center. Measurement was taken in a *θ*–2*θ* geometry from 10° to 60° at 45 kV and 40 mA tube power. The (NH_4_)_2_CuCl_4_·2H_2_O crystals were determined to have a tetragonal structure with the lattice constants *a* = *b* = 7.5991 Å, *c* = 7.9661 Å, *α* = *β* = *γ* = 90°. This result is consistent with those of previous studies.^1,17^ The DSC analysis of the (NH_4_)_2_CuCl_4_·2H_2_O crystals revealed three endothermic peaks during heating, as shown in Fig. 2. The mass of the powdered samples used in the DSC experiment is 6.6 mg. The endothermic peak enlarged in Fig. 2 near 200 K (=*T*_C1_) is consistent with the phase transition temperature reported previously, and is very small relative to the other endothermic peaks. The endothermic peaks near 406 K (=*T*_C2_) and 437 K (=*T*_C3_) are related to the thermal dehydration according to the below-mentioned loss of H_2_O. TG analysis was used to determine whether these high-temperature transformations were structural phase transitions or melting temperatures. The TG curve of (NH_4_)_2_CuCl_4_·2H_2_O is shown in Fig. 3. The first occurrence of mass loss begins at approximately 364 K, and at 406 K, 96.75% of mass remains, yielding (NH_4_)_2_CuCl_4_·1.5H_2_O. The mass loss at 430 K and 437 K reaches 6.49% and 9.74% for (NH_4_)_2_CuCl_4_·H_2_O and (NH_4_)_2_CuCl_4_·0.5H_2_O, respectively. The bulk mass of (NH_4_)_2_CuCl_4_·2H_2_O decreases at 364 K (*T*_d_), which is ascribed to the onset of partial thermal decomposition, and reaches complete thermal decomposition, thereby turning into (NH_4_)_2_CuCl_4_, at approximately 443 K. The transformation anomalies at 406 (=*T*_C2_) and 437 K (=*T*_C3_) in the DSC experiment are related to the phase transitions from (NH_4_)_2_CuCl_4_·2H_2_O to (NH_4_)_2_CuCl_4_·1.5H_2_O and from (NH_4_)_2_CuCl_4_·2H_2_O to (NH_4_)_2_CuCl_4_·0.5H_2_O, respectively. Optical polarizing microscopy showed that the crystals are light blue in color at room temperature and that they undergo color changes as the temperature increases. As the temperature increases, the color of the crystal varies from light blue (295 K and 373 K) to light green (386 K) to green (393 K) to dark yellow (433 K) and finally to brown (448 K), as shown in the inset of Fig. 3. This color change may be related to the partial loss of H_2_O. The DSC peaks at 406 K and 437 K are related to chemical changes through thermal dehydration, based on the TG and optical polarizing microscopy results. The weight loss evidenced by the TG curve suggests that *T*_C2_ and *T*_C3_ in (NH_4_)_2_CuCl_4_·2H_2_O are not related to physical changes such as structural phase transitions. Rather, they are related to a chemical change through thermal dehydration.

**Fig. 4:**
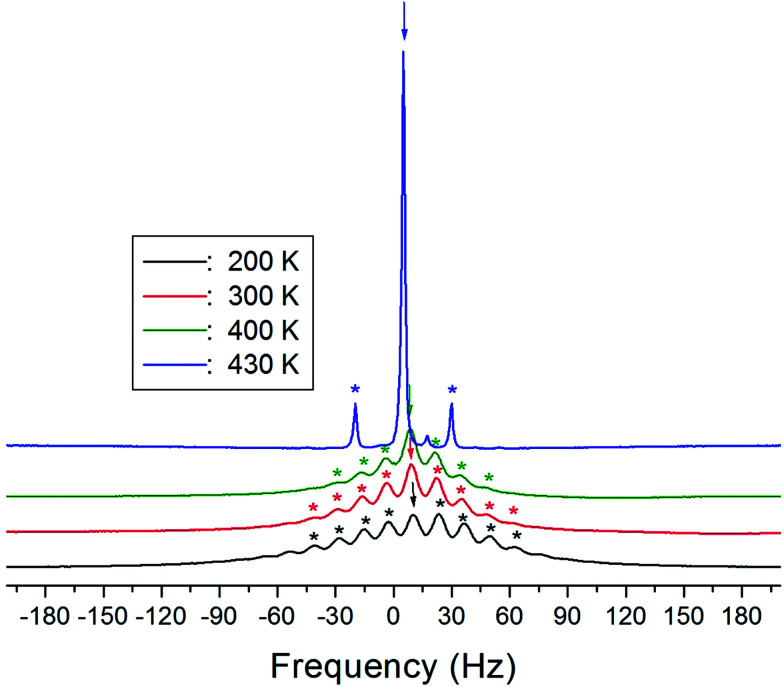
^1^H MAS NMR spectrum of (NH_4_)_2_CuCl_4_·2H_2_O at several temperatures.

The ^1^H MAS NMR spectra of (NH_4_)_2_CuCl_4_·2H_2_O, which were obtained as a function of temperature, only exhibit one peak ascribed to chemical shift, as shown in Fig. 4. The spinning sidebands of the peak are marked with asterisks. There are two kinds of protons in (NH_4_)_2_CuCl_4_·2H_2_O: ammonium protons and water protons. The current experiment was unable to distinguish the signals resulting from these two types of protons, because the eight protons from ammonium and the four protons from water are expected to yield two superimposed lines. Thus, the signal generated by the ammonium protons might include the signal caused by the water protons.

**Fig. 5:**
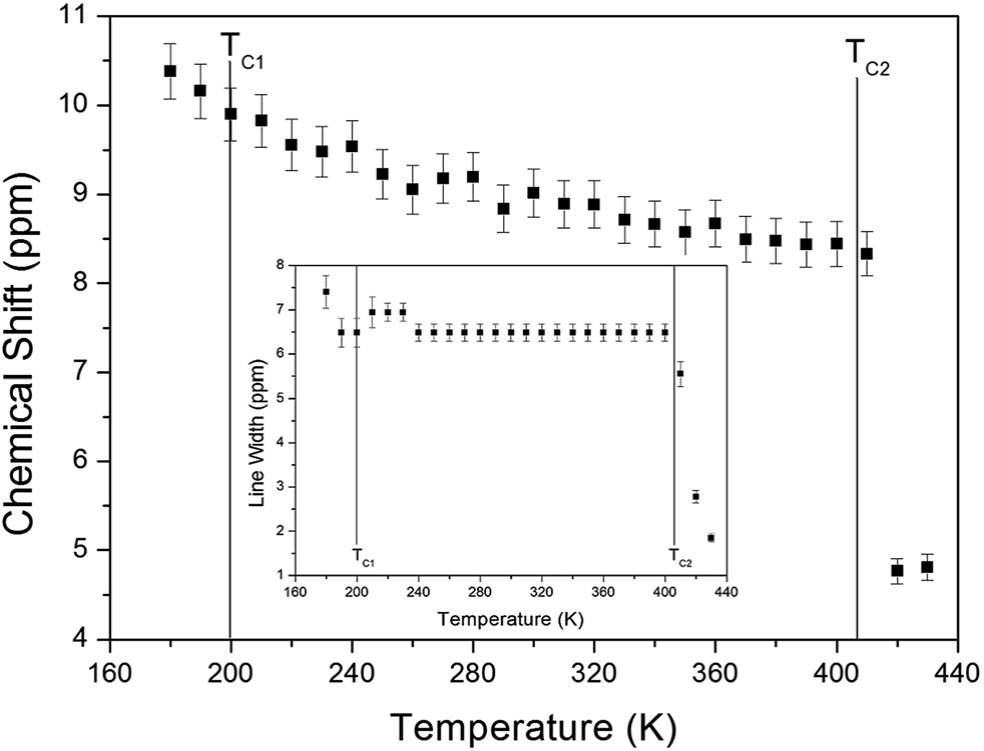
Chemical shift of ^1^H MAS NMR spectrum of (NH_4_)_2_CuCl_4_·2H_2_O as a function of temperature. (inset: line width of the ^1^H MAS NMR spectrum of (NH_4_)_2_CuCl_4_·2H_2_O).

The chemical shifts for the ^1^H nuclei in (NH_4_)_2_CuCl_4_·2H_2_O with respect to tetramethylsilane (TMS) at a frequency of 400.13 MHz are presented in Fig. 5 as a function of temperature. The chemical shifts of the ^1^H nucleus change abruptly near *T*_C2_, whereas those near *T*_C1_ are continuous. The change in the chemical shift with temperature indicates that the configuration of the atoms neighboring the ^1^H nuclei is undergoing change. The full width at half maximum (FWHM) of the ^1^H MAS NMR signal is shown in the inset of Fig. 5 as a function of temperature. As the temperature increases, the FWHM near *T*_C1_ is continuous, and that near *T*_C2_ decreases in a step-like shape. This stepwise narrowing is generally considered to be caused by internal motions, which have a temperature dependence related to that observed for the chemical shift. The shape of the line changes progressively with increasing temperature from the Gaussian-like shape of a rigid lattice to a Lorentzian shape. Near *T*_C2_ the line width undergoes an abrupt drop, after which the line width becomes considerably narrower.

The nuclear magnetization recovery traces for ^1^H MAS NMR are usually represented by a single-exponential function. However, the magnetization recovery traces for ^1^H MAS NMR in (NH_4_)_2_CuCl_4_·2H_2_O could be described by the following double-exponential function:^18–20^

**Fig. 6:**
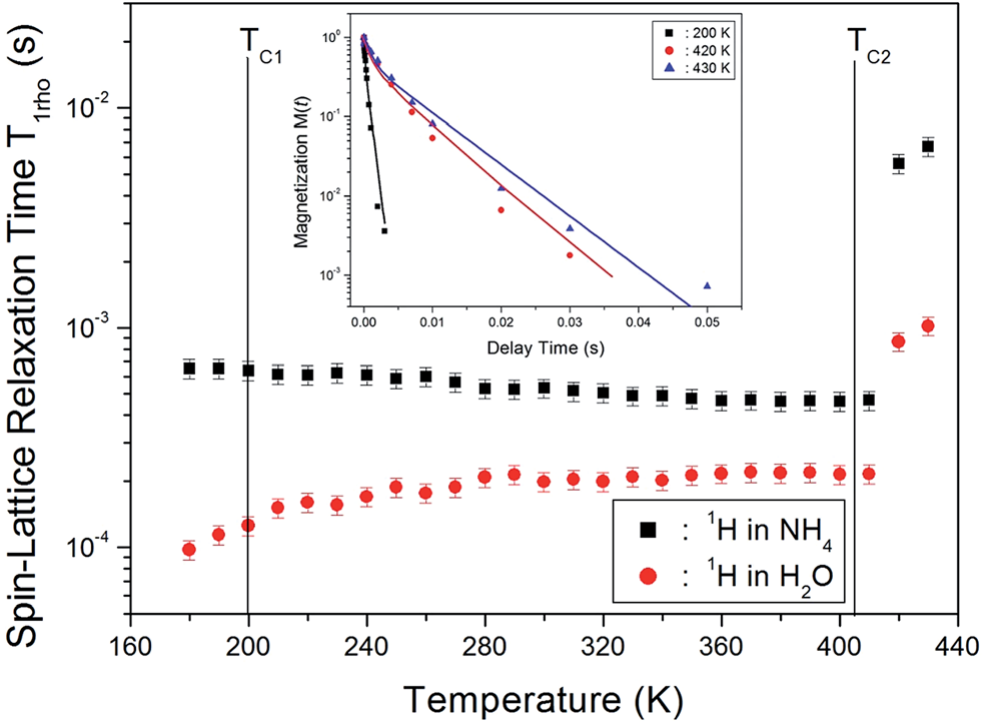
Temperature dependences of the ^1^H spin-lattice relaxation time in the rotating frame, *T*_1ρ_(s) and *T*_1ρ_(l) for H_2_O and NH_4_ in (NH_4_)_2_CuCl_4_·2H_2_O (inset: the saturation recovery traces for delay time for ^1^H in (NH_4_)_2_CuCl_4_·2H_2_O).

1 M(*t*)/M(∞) = *a* exp(−*t*/*T*_1ρ_(s)) + *b* exp(−*t*/*T*_1ρ_(l)),

where *T*_1ρ_(s) and *T*_1ρ_(l) are the short and long spin-lattice relaxation times, respectively. The magnetization recovery traces showing the delay time of the ^1^H resonance signal at temperatures of 200 K, 420 K, and 430 K are shown in the inset of Fig. 6. The obtained spin-lattice relaxation curves were well fitted by the abovementioned double-exponential function, with the slope of the recovery trace decreasing as the temperature increased. Note that the occurrence of a double-exponential spin-lattice relaxation pattern is unusual for a strongly dipolar-coupled proton system, whereas spin diffusion is expected to afford a single-exponential relaxation pattern.^21^ Therefore, we concluded that the proton system comprises two spatially well-separated nuclei. The values of *T*_1ρ_ for ^1^H in (NH_4_)_2_CuCl_4_·2H_2_O at several temperatures are shown in Fig. 6. As mentioned above, two different sets of *T*_1ρ_ values were obtained from the double-exponential function: the larger values, *T*_1ρ_(l), correspond to the longer N–H⋯Cl chain systems in the NH_4_ groups, whereas the smaller ones, *T*_1ρ_(s), correspond to the shorter O–H⋯Cl chain systems in the H_2_O groups. Here, *T*_1ρ_(l) decreases slightly with increasing temperature, as opposed to *T*_1ρ_(s), which increases with increasing temperature. Furthermore, *T*_1ρ_(s) and *T*_1ρ_(l) for ^1^H in these groups were continuous in close proximity to *T*_C1_, whereas *T*_1ρ_(s) and *T*_1ρ_(l) of ^1^H in both the H_2_O and NH_4_ groups were discontinuous close to *T*_C2_, corresponding to an abrupt increase with increasing temperature. The abrupt rise in *T*_1ρ_ near *T*_C2_ contributes to the increased mobility of the protons in H_2_O and NH_4_. The changes observed for the ^1^H nucleus near *T*_C2_ are related to variations in the symmetry of the environments of H, *i.e.*, to changes in the symmetry of the NH_4_ and H_2_O groups. The forms of the tetrahedrons of water molecules are probably disrupted by the loss of H_2_O.

**Fig. 7:**
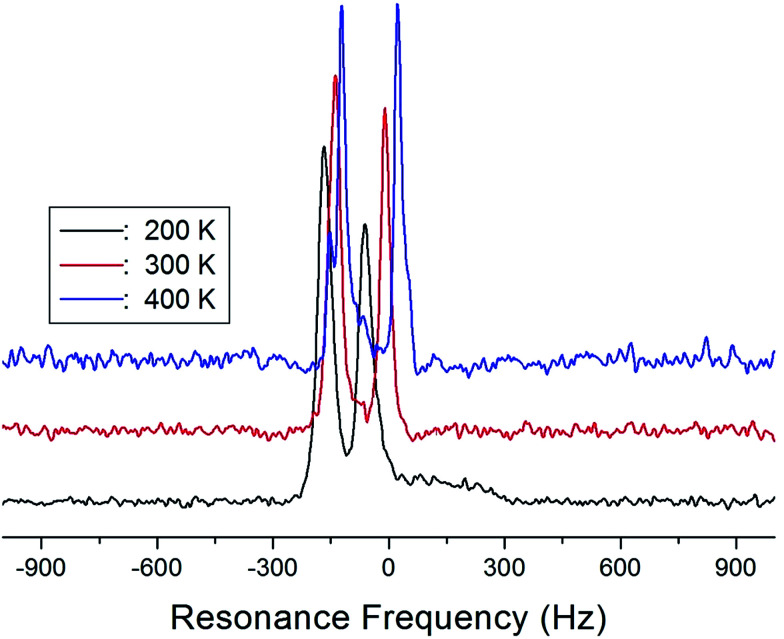
^14^N NMR spectrum of a (NH_4_)_2_CuCl_4_·2H_2_O single crystal at 200 K, 300 K, and 400 K.

**Fig. 8:**
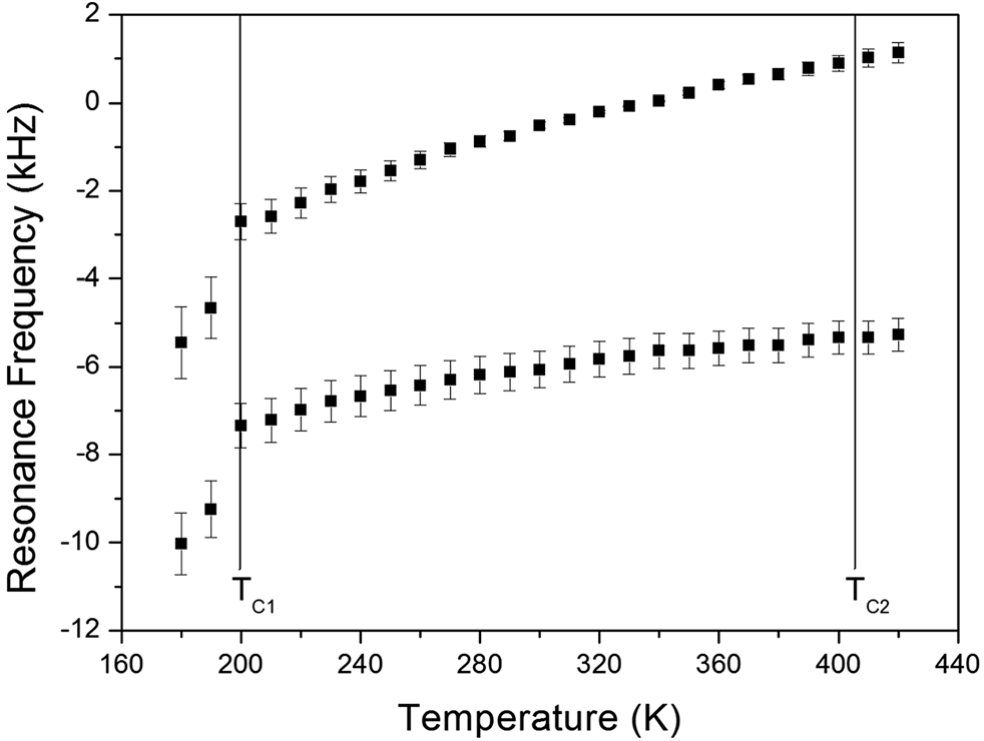
Resonance frequencies of ^14^N NMR spectrum of a (NH_4_)_2_CuCl_4_·2H_2_O single crystal as a function of temperature.

The NMR spectra of ^14^N of (NH_4_)_2_CuCl_4_·2H_2_O single crystals were recorded in order to investigate local phenomena related to the phase transition. The ^14^N nucleus has a spin number 1. Spectra were obtained by the solid-state echo method using static NMR at a Larmor frequency of *ω*_0_/2π = 43.342 MHz in the laboratory frame. The ^14^N NMR spectra of (NH_4_)_2_CuCl_4_·2H_2_O single crystals at 200, 300, and 400 K are shown in Fig. 7. Two resonance lines are expected because of the quadrupole interaction of the ^14^N (*I* = 1) nucleus.^19^ With respect to the crystal orientation, the magnetic field was applied along the crystallographic *c*-axis. The resonance frequencies of the ^14^N signals are plotted in Fig. 8, as a function of temperature. In the vicinity of *T*_C1_, the frequencies of both signals are discontinuous, whereas those near *T*_C2_ are continuous, and the resonance frequency increases with increasing temperature. In addition, the splitting of the ^14^N resonance lines above 200 K increases slightly with increasing temperature. These temperature-dependent changes in the ^14^N resonance frequencies are attributed to changes in the structural geometry of the NH_4_^+^ ion.^22^ In all temperature, all nitrogen is physically equivalent, and the ^14^N quadrupole parameter is slowly increased.^23^ In this case, the electric field gradient (EFG) tensors at the N sites are varied, reflecting the changing atomic configurations around the ^14^N nuclei in NH_4_.

## IV. Conclusion

The thermodynamic properties and structural mechanisms near the phase transition temperatures in (NH_4_)_2_CuCl_4_·2H_2_O were studied through DSC, TG, NMR chemical shift, and the spin-lattice relaxation time *T*_1ρ_. The DSC and TG results indicate that the endothermic peak near 200 K (=*T*_C1_) is a structural phase transition, and the endothermic peaks near 406 K (=*T*_C2_) and 437 K (=*T*_C3_) are related to a chemical change through thermal dehydration due to the escape of H_2_O. On the other hand, the changes in the temperature dependence of the chemical shift, linewidth, and spin-lattice relaxation time *T*_1ρ_ near *T*_C2_ are related to variations in the symmetry of the environments of NH_4_ and H_2_O. The mechanism of these changes at high temperature is related to hydrogen bond proton transfer involving the breakage of a weak hydrogen bond. The change at *T*_C2_ and *T*_C3_ seems to be a chemical change caused by thermal decomposition rather than a physical change such as a structural phase transition, and the tetrahedrons formed by the water molecules are probably disrupted by the loss of H_2_O. From the DSC, TG, and NMR data, it is clear that the structural change at high temperature arises due to the loss of the two water molecules coordinated to the Cu^2+^ ion along the *c*-axis.

## Conflicts of interest

There are no conflicts to declare.

## Supplementary Material

## References

[cit1] Narsimlu N., Kumar K. S. (2002). Cryst. Res. Technol..

[cit2] Tylczynski Z., Wiesner M., Trzaskowska A. (2016). Phys. B.

[cit3] Lim A. R., Cho J. (2017). J. Mol. Struct..

[cit4] Klaassen T. O., Poulis N. J. (1971). J. Phys. Colloq..

[cit5] Zavodnik V. E., Bel'skii V. K., Diaz I., Fernandez V. (1999). Crystallogr. Rep..

[cit6] Kaminsky W., Haussuhl S., Brandstadtor A., Balarew C. (1994). Z. Kristallogr..

[cit7] Franke V. D., Punin Y. O., P'yankova L. A. (2007). Crystallogr. Rep..

[cit8] Czlonkowska M., Tylczynski Z., Laniecki M. (2006). Cryst. Res. Technol..

[cit9] Stefov V., Soptrajanov B. (1999). Vib. Spectrosc..

[cit10] Bhakay-Tamhane S. N., Sequeira A., Chidanbaram R. (1980). Acta Crystallogr., Sect. B: Struct. Crystallogr. Cryst. Chem..

[cit11] Chidambaram R., Navarro Q. O., Garcia A., Linggoatmodjo K., Chien L. S., Suh I.-H. (1970). Acta Crystallogr., Sect. B: Struct. Crystallogr. Cryst. Chem..

[cit12] Waizumi K., Masuda H., Ohtaki H. (1992). Acta Crystallogr., Sect. C: Cryst. Struct. Commun..

[cit13] Tylczynski Z., Wiesner M. (2015). Mater. Chem. Phys..

[cit14] bansal M. L., Sahni V. C., Roy A. P. (1979). J. Phys. Chem. Solids.

[cit15] Suga H., Sorai M., Yamanaka T., Seki S. (1965). Bull. Chem. Soc. Jpn..

[cit16] Narsimlu N., Sivakumar K., Sastry G. S. (1996). Cryst. Res. Technol..

[cit17] Bhakay-Tamhane S. N., Sequeira A., Chidambaram R. (1980). Acta Crystallogr., Sect. B: Struct. Crystallogr. Cryst. Chem..

[cit18] Koenig J. L. , Spectroscopy of Polymers, Elsevier, New York, 1999

[cit19] Abragam A. , The Principles of Nuclear Magnetism, Oxford University Press, 1961

[cit20] Cowan B. , Nuclear Magnetic Resonance and Relaxation, Cambridge University, UK, 1997

[cit21] Lim A. R., Kim S. W., Joo Y. L. (2017). J. Appl. Phys..

[cit22] Lim A. R. (2016). Solid State Sci..

[cit23] Seliger J., Blinc R., Arend H., Kind R. (1976). Z. Phys., B, Condens. matter.

